# CALLY Index and Inflammatory Parameters for Predicting Focal Involvement in Human Brucellosis: A Retrospective Cohort Study

**DOI:** 10.3390/diagnostics16162607

**Published:** 2026-08-17

**Authors:** Mehmet Ali Tüz, Yeşim Çağlar, Enes Dalmanoğlu, Rukiye İnan Sarıkaya, Derya Tuna Ecer, İrem Tataroğlu

**Affiliations:** Department of Infectious Diseases and Clinical Microbiology, Faculty of Medicine, Balikesir University, Altieylül 10145, Balikesir, Turkey; yesim.alpay@balikesir.edu.tr (Y.Ç.); enes.dalmanoglu@balikesir.edu.tr (E.D.); rukiye.sarikaya@balikesir.edu.tr (R.İ.S.); deryatunaa@hotmail.com (D.T.E.); iremtataroglu460@hotmail.com (İ.T.)

**Keywords:** albumin, brucellosis, biomarkers, ESR, predictive value of tests, inflammation, blood sedimentation, CALLY index

## Abstract

**Background:** Focal involvement in brucellosis complicates clinical management and increases treatment failure risk. This study evaluated the predictive value of routine inflammatory markers and, for the first time, the C-reactive protein-albumin-lymphocyte (CALLY) index for predicting focal complications. **Methods:** A retrospective cohort study conducted between February 2016 and January 2026 included 203 brucellosis patients, categorized into “no focal involvement” (n = 114) and “focal involvement” (n = 89) groups. Admission blood count parameters, erythrocyte sedimentation rate (ESR), C-reactive protein (CRP), and CALLY index were compared using Receiver Operating Characteristic (ROC) curves, DeLong tests, and multivariate logistic regression models incorporating CRP, albumin, CALLY index, ESR, hemoglobin, MPV, age, and gender. **Results:** Patients with focal involvement were older (*p* = 0.029) and exhibited higher ESR, CRP, neutrophil-to-lymphocyte, and platelet-to-lymphocyte ratios but lower hemoglobin, mean platelet volume, albumin, and CALLY index (*p* < 0.001). ROC analysis showed ESR had the highest predictive value (AUC: 0.839), followed by albumin (AUC: 0.751) and the CALLY index (AUC: 0.728). DeLong tests confirmed ESR’s statistical superiority over the CALLY index (*p* = 0.002) and albumin (*p* = 0.016). In multivariate regression, ESR remained a robust independent prognostic factor (OR: 1.042, *p* < 0.001), whereas the CALLY index lacked independent significance. **Conclusions:** Despite its theoretical appeal as a novel immuno-nutritional biomarker, the CALLY index does not outperform traditional markers in predicting focal complications of brucellosis at initial presentation. ESR remains a robust, inexpensive, and statistically superior independent predictor. These findings emphasize that clinicians should continue to rely on widely accessible traditional markers like ESR for early diagnostic risk stratification, while the potential utility of composite scores like the CALLY index may be better reserved for monitoring dynamic therapeutic responses and managing chronic cases.

## 1. Introduction

Brucellosis is one of the most common zoonotic infections worldwide. According to the World Health Organization (WHO), approximately 500,000 incident human cases are reported annually; however, due to widespread misdiagnosis and underreporting, the true global incidence is estimated to be substantially higher, potentially reaching millions [1]. The disease imposes a massive socioeconomic and healthcare burden due to severe physical debilitation and livestock-related agricultural losses [2]. Consequently, brucellosis continues to remain a significant public health problem in the Mediterranean Basin, the Middle East, South America, and developing countries [3]. Brucellosis can affect all organs and systems regardless of whether the disease is in its acute or chronic phase. The specific organ involvement of the disease is termed “focal involvement” or complicated brucellosis [4]. According to studies, complication rates associated with brucellosis in endemic regions are reported across a wide range of 28.1% to 89.7%. The most frequently encountered focal involvements primarily include the osteoarticular system (spondylitis, sacroiliitis, peripheral arthritis), followed by hematological, gastrointestinal, and genitourinary system involvements [4,5,6]. The therapeutic management of patients who develop focal involvement is considerably more difficult compared to uncomplicated cases; it requires a prolonged duration of treatment and predominantly necessitates the use of triple-antibiotic combinations. Furthermore, the presence of focal involvement is an independent risk factor for therapeutic failure. Delayed or inadequate treatment can lead to permanent organ function loss, disease relapse, and, rarely, mortality in these patients [4,7].

Early diagnosis of focal involvement in brucellosis is of critical importance to prevent labor and financial losses, reduce morbidity, and avert therapeutic failure. Although the detection of complications relies on the isolation of the causative agent from blood or tissue cultures and the evaluation of organ involvement via radiological imaging, the lack of rapid results from culture methods and the occasional unavailability of imaging techniques have prompted clinicians to search for more practical biomarkers capable of predicting focal involvement at an early stage [4,6].

In recent years, the role of routinely analyzed complete blood count parameters and inflammatory markers in predicting focal involvement has drawn substantial attention. The literature has demonstrated that high erythrocyte sedimentation rates (ESR), elevated leukocyte and neutrophil counts, and increased C-reactive protein (CRP) levels may be associated with focal involvement. Some recent studies indicate that an elevated neutrophil-to-lymphocyte ratio (NLR) and platelet-to-lymphocyte ratio (PLR), decreased mean platelet volume (MPV), and high platelet counts could serve as strong indicators of organ involvement or osteoarticular complications [5,6]. Additionally, low serum albumin levels have been identified as an independent factor associated with poor clinical prognosis and therapeutic failure in focal brucellosis [4,8]. While separate studies have highlighted the individual significance of CRP, lymphocyte counts, and albumin levels in predicting complications and treatment failure, no study to date has evaluated the combined utility of these parameters.

In the current literature, the CALLY (C-reactive protein, Albumin, and Lymphocyte) index has emerged as a non-invasive and cost-effective prognostic biomarker that synthesizes the patient’s systemic inflammatory response and immuno-nutritional status into a single integrated parameter [9,10]. While various composite scores are utilized in infectious diseases, we specifically selected the CALLY index because its tri-dimensional structure simultaneously captures systemic inflammation, nutritional stress (albumin), and host cellular immunity (lymphocyte count). Compared to the isolated evaluation of single acute-phase reactants like CRP or other bi-dimensional indices, this holistic reflection of the host’s physiological state is suggested to be more beneficial in predicting the clinical trajectory of infectious processes [11,12]. The CALLY index has been investigated as a prognostic biomarker in other systemic inflammatory conditions (e.g., malignancy, acute coronary syndrome) and severe infectious diseases (e.g., infective endocarditis, sepsis). However, the clinical utility of the CALLY index in a complex, multisystemic infection like brucellosis has not yet been investigated [10,12,13,14].

The aim of this study is to evaluate the predictive value of common inflammatory markers reported in the literature for the early prediction of focal involvement in patients with brucellosis. Additionally, for the first time in the literature, we aim to investigate the predictive performance of the CALLY index in differentiating focal complications and to explore its potential utility as a novel clinical risk stratification tool.

## 2. Materials and Methods

### 2.1. Study Design and Patient Selection

**Figure 1:**
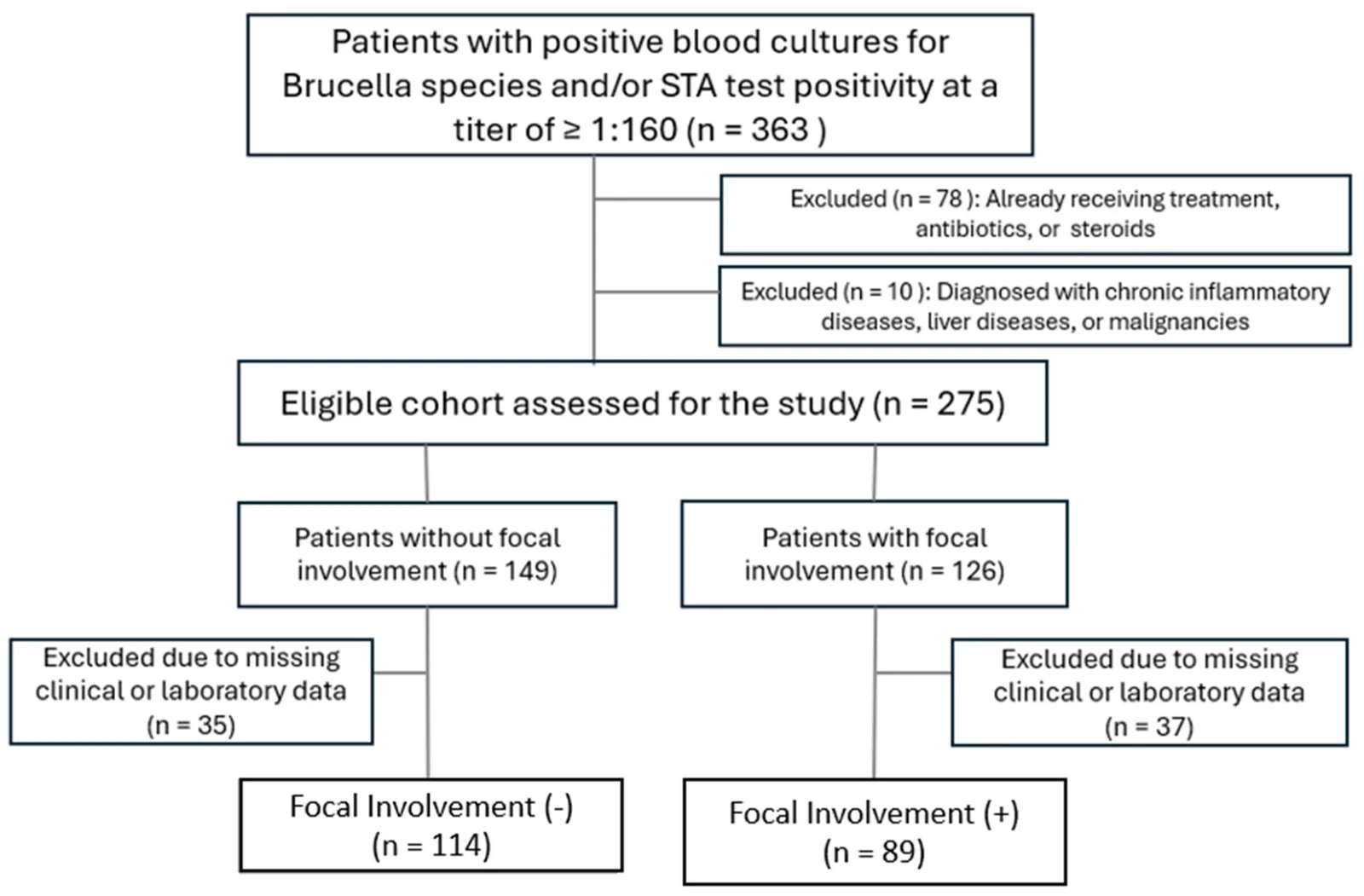
Study flowchart.

This retrospective study was conducted by analyzing the data of brucellosis patients followed and treated at the Infectious Diseases and Clinical Microbiology Clinic of Balikesir University Health Application and Research Hospital between 1 February 2016 and 31 January 2026. The study protocol was approved by the Health Research Ethics Committee of Balikesir University Faculty of Medicine on 17 March 2026 (Protocol No: 2026/6-4). All consecutive patients meeting the inclusion criteria were enrolled. The sample size was determined by the available patient records during the study period. A total of 203 brucellosis patients were included, and they were classified into two main groups based on clinical, radiological, and laboratory findings: “No Focal Involvement (-)” (n = 114) and “Focal Involvement (+)” (n = 89). Figure 1 shows the flowchart for this study.

Patients under 18 years of age, those with chronic inflammatory diseases, liver disease, malignancy, or concurrent active infections, individuals receiving immunosuppressive therapy, and those with a history of antibiotic or corticosteroid use within the preceding three months were excluded from the study. Blood samples were collected at initial presentation, strictly prior to the initiation of antimicrobial therapy. Furthermore, patients with incomplete data for the evaluated laboratory parameters were excluded from the study.

### 2.2. Diagnostic Criteria and Definition of Focal Involvement

The diagnosis of brucellosis was established by the presence of compatible clinical symptoms (e.g., fever, sweating, arthralgia) accompanied by the isolation of *Brucella* species from blood cultures and/or standard tube agglutination (STA) test positivity at a titer of ≥1:160. In accordance with the established literature, the clinical phases of the disease were classified based on the duration of symptoms prior to diagnosis: the acute phase was defined as a symptom duration of less than 8 weeks, whereas the subacute/chronic phase was defined as a symptom duration of 8 weeks or longer.

Clinical complications developing during the disease course were categorized as osteoarticular, hematological, gastrointestinal, genitourinary, neurological, cardiovascular, ocular, and dermatological involvements. In accordance with methodological standards in the literature, all specific organ and anatomical region infections—excluding hematological variations representing isolated systemic laboratory abnormalities—were defined as “focal involvement” or “focal complications”. All diagnostic criteria were applied uniformly by two infectious disease specialists. Due to the retrospective observational design of the study, a uniform radiological screening protocol was not universally applied to all patients. Instead, imaging modalities (e.g., MRI, CT, USG) were symptom-driven and strictly performed on patients exhibiting specific clinical signs suggestive of focal complications to confirm the diagnosis. The clinical, laboratory, and radiological diagnoses of focal organ involvements were based on the following objective criteria:

- **Osteoarticular Involvement:** Defined by the presence of objective inflammatory signs (localized pain, tenderness, swelling, erythema, and restricted joint movement) in the affected joint or spinal segment; combined with pathological radiological findings compatible with brucellosis (spondylodiscitis, sacroiliitis, peripheral arthritis, bursitis, enthesitis, or paravertebral/psoas abscess) demonstrated via plain radiography, computed tomography (CT), or magnetic resonance imaging (MRI).
- **Gastrointestinal Involvement:** Investigated in patients presenting with symptoms such as nausea, vomiting, diarrhea, constipation, or abdominal tenderness/pain. Hepato-splenomegaly was documented via abdominal ultrasonography (USG). Hepatic parenchymal involvement was defined as an at least 5-fold increase above the upper normal limit in serum alanine aminotransferase (ALT) and aspartate aminotransferase (AST) levels, in the absence of any other primary etiology capable of causing hepatic dysfunction [6].
- **Genitourinary Involvement:** Encompassed cases of epididymo-orchitis presenting with acute scrotal/testicular pain, swelling, local tenderness, hyperemia, and edema on clinical examination, which were radiologically confirmed by scrotal USG revealing epididymitis, orchitis, or scrotal abscess formation.
- **Neurological Involvement (Neurobrucellosis):** Defined as the laboratory confirmation of meningoencephalitis through the isolation of *Brucella* spp. in cerebrospinal fluid (CSF) analysis and/or standard tube agglutination (STA) test positivity (at any titer) in the CSF samples of patients exhibiting acute or chronic neurological/psychiatric clinical findings compatible with brucellosis.
- **Dermatological Involvement:** Skin lesions not attributable to an underlying primary cutaneous pathology or allergic reaction, demonstrating clinical regression upon specific anti-brucellosis combination therapy, and accompanying the systemic course of the disease were classified as focal dermatological involvement.

The ‘no focal involvement’ group was operationally defined as patients who presented with systemic symptoms of brucellosis but lacked any clinical, microbiological, or radiological evidence satisfying the aforementioned criteria for focal complications.

### 2.3. Data Collection and Laboratory Analysis

Patients’ demographic characteristics (age, sex), epidemiological risk factors (animal husbandry, consumption of unpasteurized dairy products), and clinical presentation upon admission (fever, headache, bacteremia) were extracted from the hospital information management system. Complete blood count parameters (leukocytes, monocytes, lymphocytes, neutrophils, platelets, hemoglobin, MPV) and biochemical markers (albumin, CRP, AST, ALT, ESR) were recorded from venous blood samples collected at admission. CRP values below the detection limit were assigned 0.1 mg/L.

The CALLY index, reflecting systemic inflammation and nutritional status, was calculated using the following explicitly defined formula:

$$\mathrm{CALLY}\;\mathrm{Index}=\frac{\mathrm{Serum}\;\mathrm{albumin}\;(g/L)\times \mathrm{Lymphocyte}\;\mathrm{count}\;(10^{3}/\mu L)}{\mathrm{CRP}\;(\mathrm{mg}/L)}$$

Additionally, the Neutrophil-to-Lymphocyte Ratio (NLR), Platelet-to-Lymphocyte Ratio (PLR), and Lymphocyte-to-Monocyte Ratio (LMR) were computed utilizing standard formulas.

Blood cultures were analysed using the BD BACTEC FX blood culture system (Becton Dickinson, Franklin Lakes, NJ, USA). *Brucella* spp. were identified using conventional methods. The serum agglutination test was performed using *Brucella abortus* S.99 antigen. White blood cell count, neutrophil count, lymphocyte count, platelet count and MPV were measured using a Sysmex XN-1000 haematology analyser (Sysmex Corporation, Kobe, Japan).

### 2.4. Statistical Analysis

Statistical evaluation was conducted using SPSS version 25.0 (IBM Corporation, Armonk, NY, USA) and R-based software, version 4.3.2 (R Foundation for Statistical Computing, Vienna, Austria). Specifically, the ‘pROC’ package was utilized within the R environment to perform the ROC curve analysis and the DeLong test. The normality of continuous variables was assessed; normally distributed continuous variables were expressed as Mean ± Standard Deviation (SD), and non-normally distributed continuous variables as Median and Interquartile Range (IQR). The Shapiro–Wilk and Kolmogorov–Smirnov tests were used to analyse the normality of continuous variables. For intergroup comparisons, Student’s *t*-test, the Mann–Whitney U test, and the Chi-square test were utilized. Receiver Operating Characteristic (ROC) curve analysis was performed to determine the diagnostic performance of laboratory parameters in predicting focal involvement, calculating the Area Under the Curve (AUC), sensitivity, and specificity values. Optimal cut-off values were determined using the Youden index. The DeLong test was employed to compare the AUCs of laboratory parameters. Variables with a univariate *p*-value < 0.25 or established clinical relevance were selected for multivariate analysis, following a multicollinearity check (*r* > 0.40). To prevent inherent mathematical coupling, the CALLY index was never modeled alongside its constituent parameters (CRP and albumin). Instead, two distinct logistic regression models were constructed to evaluate CRP and the CALLY index separately, with their AUCs compared via the DeLong test. Model calibration was evaluated using the Hosmer–Lemeshow goodness-of-fit test. Furthermore, internal validation of the multivariate logistic regression models was performed using a bootstrapping technique with 1000 resamples to confirm the robustness and internal validity of the predictive estimates. To ensure the reliability of the logistic regression models and prevent overfitting, the rule of ten events per variable (EPV) was observed. Given the 89 events (patients with focal involvement) in our dataset, Model 1 and Model 2 included 7 and 6 predictors, yielding EPV ratios of 12.7 and 14.8, respectively. Both ratios comfortably exceed the recommended minimum threshold of 10, indicating adequately powered models. A *p*-value of <0.05 was considered statistically significant.

## 3. Results

### 3.1. Demographic and Clinical Characteristics

Of the 203 brucellosis patients included in the study, focal involvement was not detected in 114 (56.1%), whereas 89 (43.9%) presented with focal complications. Within the entire cohort, osteoarticular involvement was observed in 38.9% (n = 79), gastrointestinal involvement in 19.2% (n = 39), genitourinary involvement in 7.3% (n = 15), neurological involvement in 1.5% (n = 3), and cutaneous involvement in 0.9% (n = 2) of cases. In 49 patients, multiple focal involvements were observed. The mean age of the focal involvement (+) group (51.27 ± 15.37 years) was statistically significantly higher than that of the focal involvement (-) group (46.38 ± 16.13 years) (*p* = 0.029). There were no significant differences between the two groups regarding gender distribution or risk-associated exposure history (animal husbandry, fresh cheese consumption) (*p* > 0.05). Clinical evaluation upon admission revealed that a history of high fever (55.1% vs. 32.5%; *p* = 0.001) and headache (13.5% vs. 5.3%; *p* = 0.041) were significantly more prevalent among patients with focal involvement. Bacteremia rates were comparable between the groups (*p* = 0.299) (Table 1).

### 3.2. Laboratory Parameters and CALLY Index

Evaluación of laboratory findings at admission demonstrated that ESR (43 vs. 16.5 mm/h; *p* < 0.001), CRP (35 vs. 18 mg/L; *p* < 0.001), leukocyte (7.2 vs. 6.4 × 10^9^/L; *p* = 0.003), monocyte (0.6 vs. 0.5 × 10^9^/L; *p* = 0.001), neutrophil (4.1 vs. 3.5 × 10^9^/L; *p* = 0.006) and platelet counts (300.24 ± 101.14 vs. 245.15 ± 67.13 × 10^9^/L; *p* < 0.001) were significantly elevated in the focal involvement (+) group compared to the focal involvement (-) group. In contrast, hemoglobin (12.72 ± 1.489 vs. 13.74 ± 1.733 g/dL; *p* < 0.001), MPV (8.2 vs. 8.55; *p* = 0.007), and albumin levels (37 vs. 42 g/L; *p* < 0.001) were significantly lower in patients presenting with focal involvement. Analysis of inflammatory indices revealed that NLR (1.82 vs. 1.56; *p* = 0.019) and PLR (0.137 vs. 0.112; *p* = 0.001) were significantly higher, whereas LMR (3.48 vs. 4.25; *p* = 0.001) was significantly lower in the focal involvement group. The CALLY index was significantly reduced in the focal involvement (+) group [Median: 2.35 (1.00–4.43)] compared to the focal involvement (-) group [Median: 5.25 (3.03–10.92)] (*p* < 0.001) (Table 2).

### 3.3. ROC Curve Analysis and Model Comparisons (DeLong Test)

**Figure 2:**
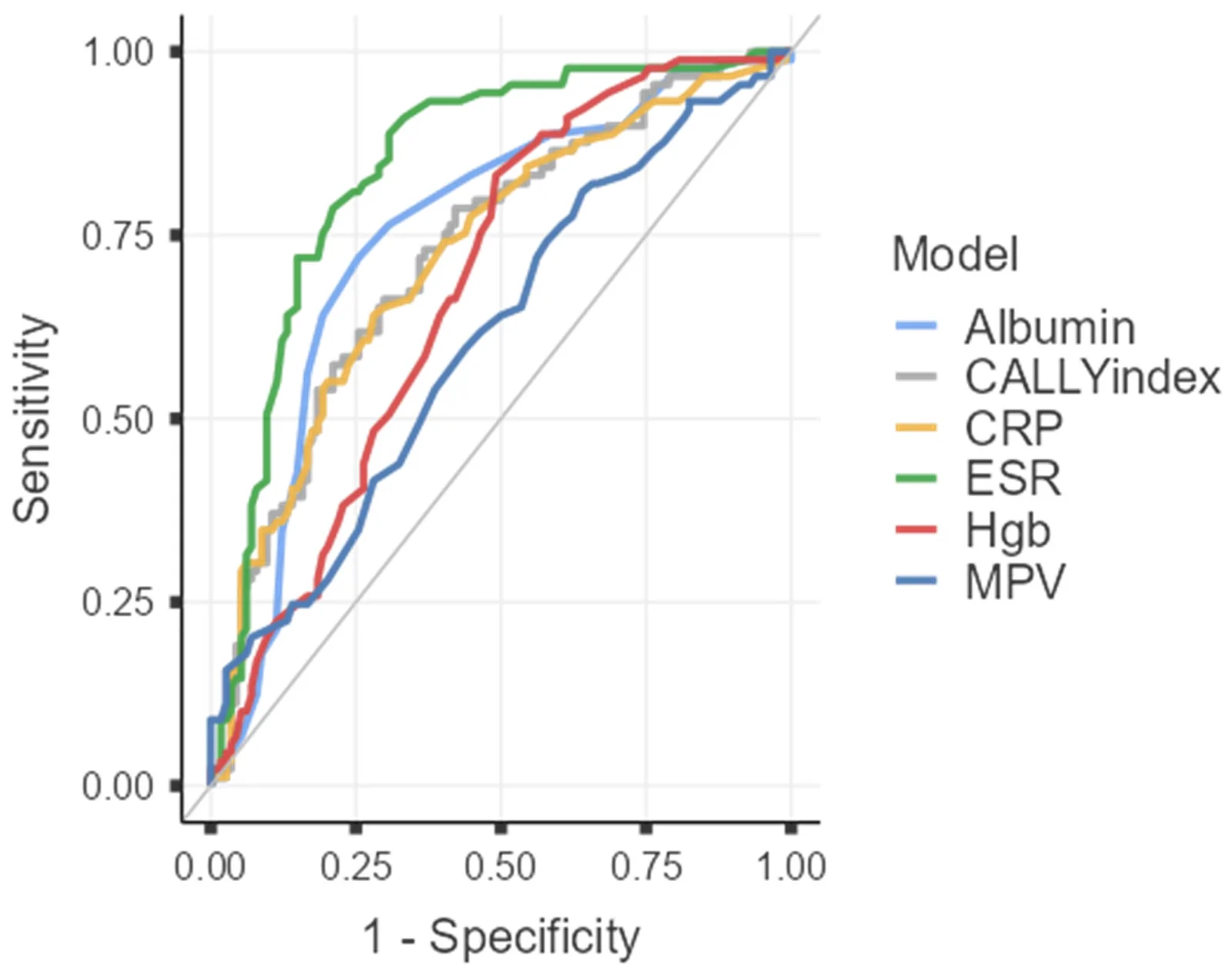
ROC curves of individual laboratory parameters for predicting focal involvement. Note: The graph illustrates the discriminative ability of various individual biomarkers in predicting the presence of focal involvement. The diagnostic performance of each parameter is represented by the Area Under the Curve (AUC) along with its 95% Confidence Interval (CI). The diagonal dashed line indicates the reference line of a random classifier with no discriminative value (AUC = 0.50). Abbreviations: AUC, Area Under the Curve; CALLY: C-reactive protein, Albumin, and Lymphocyte index; CI, Confidence Interval; CRP, C-reactive Protein; ESR, Erythrocyte Sedimentation Rate; Hgb, Hemoglobin MPV, Mean Platelet Volume; ROC, Receiver Operating Characteristic.

**Figure 3:**
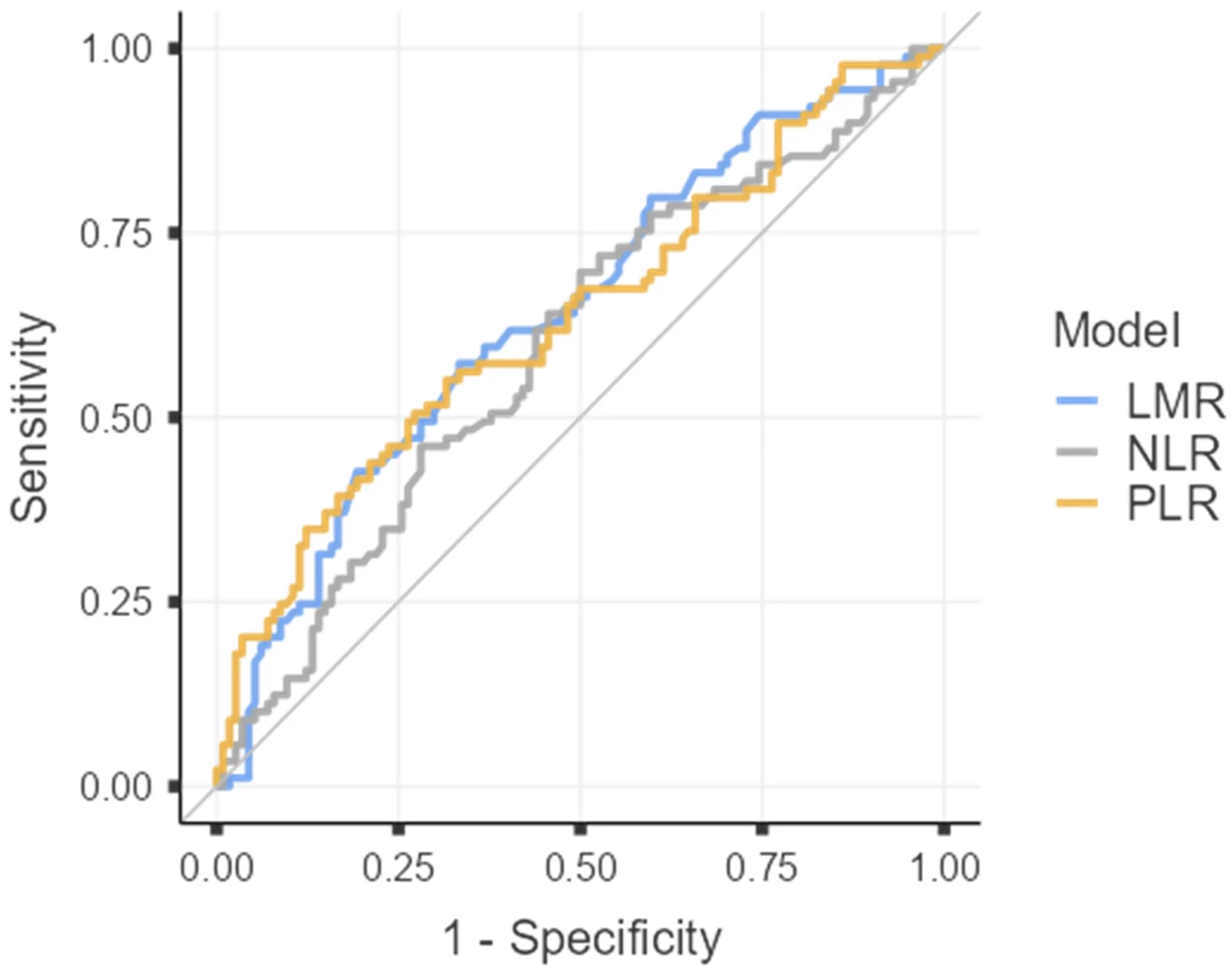
ROC curves of NLR, PLR and LMR for predicting focal involvement. Note: The graph illustrates the discriminative ability of various individual biomarkers in predicting the presence of focal involvement. The diagnostic performance of each parameter is represented by the Area Under the Curve (AUC) along with its 95% Confidence Interval (CI). The diagonal dashed line indicates the reference line of a random classifier with no discriminative value (AUC = 0.50). Abbreviations: AUC, Area Under the Curve; CALLY: C-reactive protein, Albumin, and Lymphocyte index; CI, Confidence Interval; LMR: Lymphocyte-to-Monocyte Ratio; NLR: Neutrophil-to-Lymphocyte Ratio; PLR: Platelet-to-Lymphocyte Ratio; ROC, Receiver Operating Characteristic.

Receiver Operating Characteristic (ROC) curve analysis was executed to evaluate the diagnostic accuracy of laboratory parameters in predicting focal involvement (Figure 2 and Figure 3). Among the assessed parameters, the erythrocyte sedimentation rate (ESR) exhibited the highest predictive value (AUC: 0.839, 95% CI: 0.783–0.896, *p* < 0.001). A designated cut-off value of 23.5 for ESR yielded a sensitivity of 88.76% and a specificity of 69.30%. Albumin demonstrated the second-highest diagnostic value with an AUC of 0.751 (95% CI: 0.682–0.820, *p* < 0.001), whereas the CALLY index achieved an AUC of 0.728 (95% CI: 0.658–0.798, *p* < 0.001). Utilizing an optimal cut-off value of 4.673 for the CALLY index provided 78.65% sensitivity and 57.89% specificity in identifying focal involvement. The AUC for CRP was 0.720 (95% CI: 0.649–0.791, *p* < 0.001) (Table 3).

A comparative analysis of the diagnostic capabilities of these parameters was performed using the DeLong test. The predictive performance of ESR was statistically superior to both the CALLY index (AUC Difference: 0.11443, *p* = 0.002) and Albumin (AUC Difference: 0.09087, *p* = 0.016). There was no statistically significant difference in discriminative ability between the CALLY index and CRP (AUC Difference: 0.00591, *p* = 0.678) (Table 4).

### 3.4. Multivariate Logistic Regression Analysis

**Figure 4:**
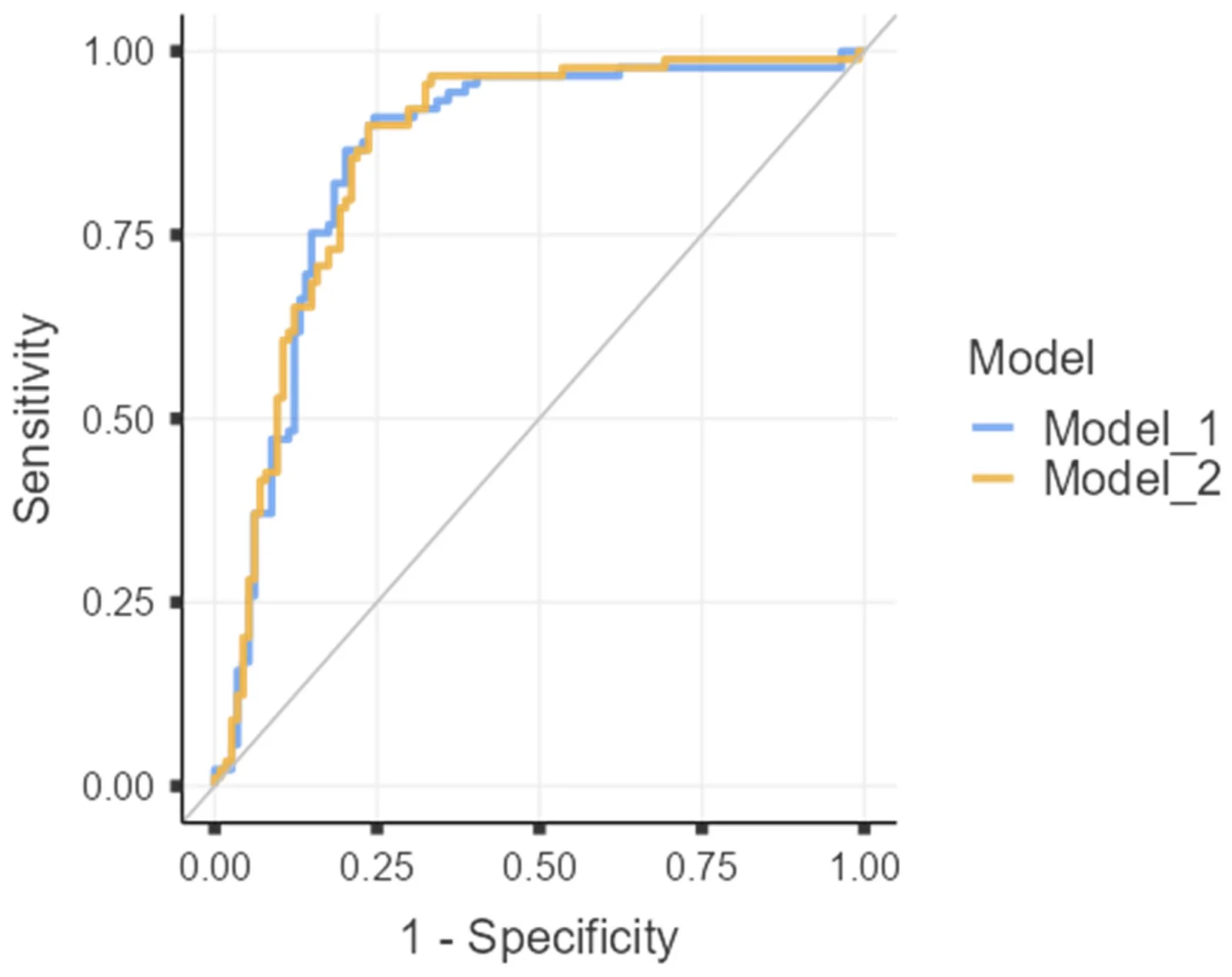
ROC curves for multivariate logistic regression models. Note: The graph displays the discriminative ability of the two distinct multivariate logistic regression models. Model 1 incorporates individual inflammatory and hematological parameters alongside demographic data (ESR, Albumin, CRP, Hemoglobin, MPV, Age, and Gender). Model 2 evaluates the logarithmic transformation of the CALLY index (LogCALLY) alongside ESR, Hemoglobin, MPV, Age, and Gender, specifically substituting CRP and Albumin to prevent inherent mathematical coupling and multicollinearity. The Area Under the Curve (AUC) reflects the overall predictive accuracy of each constructed model. The diagonal dashed line represents the reference line for a random classifier with no discriminative value (AUC = 0.50). Abbreviations: AUC, Area Under the Curve; CALLY, C-reactive protein, Albumin, and Lymphocyte; CRP, C-reactive Protein; ESR, Erythrocyte Sedimentation Rate; LogCALLY, Logarithmic transformation of the CALLY index; MPV, Mean Platelet Volume; ROC, Receiver Operating Characteristic.

To identify independent predictive factors for focal involvement, two distinct multivariate logistic regression models incorporating clinical and laboratory variables were constructed. The initial model (Model 1) evaluated CRP, Albumin, ESR, Hemoglobin, MPV, Age, and Gender, while the subsequent model (Model 2) analyzed LogCALLY, ESR, Hemoglobin, MPV, Age, and Gender. In both predictive models, ESR emerged as an independent and statistically highly significant prognostic factor for focal involvement (Model 2: OR 1.042, 95% CI 1.022–1.062, *p* < 0.001). The independent effect of the LogCALLY index, as evaluated in Model 2, did not reach statistical significance (OR 0.753, 95% CI 0.526–1.079, *p* = 0.122) (Table 5). In the evaluation of model calibration, the Hosmer–Lemeshow goodness-of-fit test yielded *p* < 0.001 for both models. While this indicates a deviation in perfect calibration—a well-documented artifact in the Hosmer–Lemeshow test when models include multiple continuous variables (such as age, CRP, ESR, and the CALLY index)—the internal validation via bootstrapping (1000 resamples) confirmed the stability of the models. The bootstrapped 95% confidence intervals for the independent predictors remained robust and statistically significant, confirming the internal validity of our predictive models. A DeLong test comparing the cumulative predictive power of the constructed models revealed no statistically significant difference between Model 1 (AUC: 0.857) and Model 2 (AUC: 0.862) (AUC Difference: 0.00542, *p* = 0.521). Figure 4 shows the ROC curves for the multivariate logistic regression models.

## 4. Discussion

Brucellosis poses a significant clinical challenge due to its propensity for focal organ involvement, which complicates therapeutic management, extends hospitalization, and elevates relapse risks [5,15]. In the present study, focal involvement was observed in 43.9% of cases, with the osteoarticular system being the most frequently affected site (38.9%). This rate is comparable to the findings of Demirdal et al. (42.6%, n = 230) and Zhang et al. (40%, n = 240), and higher than that reported by Copur et al. (28%, n = 139) [4,5,6]. The variation in reported rates likely reflects differences in complication definitions, referral patterns, and the proportion of chronic cases across cohorts. The high prevalence of osteoarticular lesions in our cohort mirrors the established consensus that *Brucella* species possess a distinct tropism for the musculoskeletal system, frequently manifesting as sacroiliitis, peripheral arthritis, and spondylodiscitis [16,17]. Given the severe morbidity associated with deep-tissue invasion, identifying early warning signs and reliable clinical or laboratory biomarkers remains vital to optimizing patient outcomes [18,19].

Demographic analysis demonstrated that patients with focal involvement were significantly older than those with uncomplicated brucellosis. This finding aligns with the extensive observations by Demirdal et al. and Copur et al., suggesting that age-related decline in cellular immunity, progressive structural degeneration in the osteoarticular system, and the presence of comorbidities may predispose older individuals to localized tissue infections [4,5]. However, exposure histories and gender distribution did not significantly differ between the groups, underscoring that host-specific immune and inflammatory responses play a more critical role in the pathogenesis of focal complications than the initial route of transmission [15].

Chronic intracellular infections like brucellosis trigger a sustained systemic inflammatory response, leading to the continuous hepatic synthesis of acute-phase reactants and a concomitant decrease in negative acute-phase proteins [20]. Consistent with the findings of Zhang et al. and Shi et al., ESR and CRP were significantly elevated, whereas serum albumin levels were markedly reduced in our focal involvement cohort [4,18]. Albumin is not only an indicator of nutritional status but also inversely reflects the severity of systemic inflammation [4]. Notably, while CRP was significantly elevated in our cohort (*p* < 0.001) and in the study by Zhang et al. (*p* = 0.003), it did not reach statistical significance in the reports by Copur et al. (*p* = 0.403) and Demirdal et al. (*p* = 0.254) [4,5,6]. This discrepancy may be attributable to differences in disease severity at presentation, the timing of blood sampling relative to symptom onset, or the varying proportions of acute versus chronic cases across the study populations. The higher proportion of acute-phase patients in our cohort (approximately 80%) may have contributed to the markedly elevated CRP levels observed in our focal involvement group.

In our ROC curve analysis, ESR emerged as the strongest single diagnostic parameter (AUC: 0.839), outperforming both albumin and CRP. Furthermore, the multivariate logistic regression models confirmed that ESR is a robust and independent predictor of focal involvement. This is clinically highly relevant, as ESR is an inexpensive, universally available, and stable marker reflecting the cumulative and sustained inflammatory burden, a hallmark of complicated brucellosis, particularly in osteoarticular cases [5,21]. Notably, the discriminative performance of ESR in our cohort (AUC 0.839) substantially exceeds that reported by Copur et al. (AUC 0.669) and the predictive value of age reported by Demirdal et al. (AUC 0.636), representing the highest discriminative accuracy for focal involvement prediction reported to date in the brucellosis literature [5,6]. Admittedly, ESR is inherently a nonspecific acute-phase reactant. However, its clinical utility becomes highly practical once a patient is serologically or microbiologically confirmed to have brucellosis. In this specific context, a markedly elevated ESR should serve as a critical triage ‘red flag’ within the diagnostic algorithm. Rather than diagnosing the infection itself, an elevated ESR should prompt clinicians to proactively integrate advanced imaging modalities (such as MRI or CT) into the patient’s workup to actively rule out deep-tissue focal complications, even if localizing clinical symptoms are initially subtle or absent.

The hematological response to *Brucella* infection involves complex alterations in cell lines, reflecting the balance between acute physiological stress and the host’s immune defense mechanisms. We observed significantly higher NLR and PLR, and lower LMR and MPV, in complicated cases. These dynamic shifts in peripheral blood cells are consistent with the review by Jin et al. and the findings reported by Feng et al. [16,22]. The elevation in neutrophils and platelets indicates an active, ongoing systemic inflammatory state and thrombopoietic stress, whereas the relative suppression of lymphocytes reflects the impairment of cellular immunity, which is essential for eradicating intracellular *Brucella* species [22,23].

The pathogenesis of brucellosis is deeply tied to this host immune response. *Brucella* species are facultative intracellular pathogens that primarily survive and replicate within macrophages, actively evading the host’s innate immune surveillance. Consequently, a robust cellular immune response—driven by macrophage activation and lymphocyte proliferation—is essential for controlling the infection, limiting bacterial dissemination, and preventing focal complications [24,25]. In this context, the lymphocyte count within the CALLY index serves as a crucial clinical surrogate for this underlying cellular immunity.

The CALLY index is a novel, comprehensive biomarker that simultaneously synthesizes the systemic inflammatory response (via CRP), nutritional status and physiological stress (via albumin), and cellular immune competence (via lymphocytes) into a single objective parameter [26]. In numerous studies investigating focal involvement and treatment failure in brucellosis, elevated CRP levels, decreased lymphocyte counts, and reduced serum albumin levels have individually been shown to possess predictive value [4,5,6]. In clinical conditions characterized by subacute or chronic inflammation, such as malignancies and chronic infectious diseases, the CALLY index, which combines these three distinct parameters, has shown promising prognostic performance compared to individual biomarkers [27]. Although its prognostic value has been reported in high-mortality conditions such as sepsis, infective endocarditis, and various malignancies, its clinical utility in brucellosis remained unexplored [13,27,28]. Theoretically, this background suggests that the CALLY index could offer enhanced prognostic accuracy in identifying brucellosis patients with focal complications, who are inherently at a higher risk for disease relapse and therapeutic failure.

However, contrary to these theoretical expectations, the CALLY index in our cohort did not outperform either its individual components or the traditional erythrocyte sedimentation rate (ESR) in predicting focal complications. The ROC curve analysis demonstrated that the CALLY index (AUC: 0.728) achieved a discriminative ability comparable to CRP alone (AUC: 0.720), with the DeLong test confirming no statistically significant difference between the two (AUC difference: 0.00591, *p* = 0.678). In contrast, ESR maintained the highest individual discriminative power (AUC: 0.839) and was significantly superior to both the CALLY index (*p* = 0.002) and CRP (*p* = 0.001). In our multivariate logistic regression analysis, the independent effect of the LogCALLY index did not reach statistical significance (OR: 0.753, 95% CI: 0.526–1.079, *p* = 0.122), whereas ESR remained a highly significant and robust independent predictor of focal involvement (*p* < 0.001). Furthermore, a DeLong test comparing the cumulative diagnostic accuracy of the two multivariate models revealed no statistically significant difference between Model 1 (CRP and albumin entered separately; AUC: 0.857) and Model 2 (LogCALLY as a composite; AUC: 0.862) (AUC difference: 0.00542, *p* = 0.521). This equivalence indicates that collapsing CRP, albumin, and lymphocytes into a single CALLY score does not enhance the predictive model beyond using these parameters individually. While previous studies have independently established the prognostic utility of ESR, CRP, and albumin, our study fundamentally advances the existing literature by providing the first direct, head-to-head statistical comparison between a comprehensive immuno-nutritional index and these traditional acute-phase reactants within the same clinical cohort. By utilizing rigorous comparative methodologies—specifically pairwise DeLong test comparisons and the parallel construction of dual multivariate models—we move beyond merely confirming the utility of routine markers. Instead, we provide definitive, mathematically validated evidence that the complex integration of these variables does not surpass the individual predictive threshold of ESR. This critical finding guides real-world clinical practice by proving that straightforward, single-parameter assessments (primarily ESR) remain statistically and practically superior to complex composite indices for the early risk stratification of focal brucellosis.

Several pathophysiological factors may explain the limited performance of the CALLY index in this clinical context. First, the majority of our cohort comprised acute-phase brucellosis patients (75.3–82.5%), in whom CRP elevation is the dominant and earliest laboratory abnormality. During the acute inflammatory phase, CRP rises rapidly and disproportionately compared to the more gradual decline in serum albumin and the relatively preserved lymphocyte counts. Consequently, the CALLY formula becomes heavily driven by its CRP component, effectively reducing the index to a CRP surrogate rather than a truly integrated composite marker. This interpretation is strongly supported by the absence of a significant difference between the CALLY index and CRP alone in the DeLong test (*p* = 0.678) [5,6,20,29].

Second, unlike malignancies and sepsis, where profound lymphopenia and severe hypoalbuminemia develop concurrently with CRP elevation, brucellosis, as a chronic intracellular infection, typically maintains near-normal lymphocyte counts even in complicated cases. In our cohort, lymphocyte counts did not significantly differ between the focal involvement (+) and (-) groups (*p* = 0.615), indicating that the lymphocyte component of the CALLY index contributed minimally to its discriminative capacity. This contrasts sharply with oncological settings, where lymphopenia is a hallmark of immunosuppression and the lymphocyte component of CALLY provides meaningful prognostic information [26,27,30].

Third, although serum albumin levels were significantly lower in the focal involvement group (*p* < 0.001), the absolute difference was modest (median 37 vs. 42 g/L), falling within a range that is clinically less impactful compared to the severe hypoalbuminemia typically encountered in advanced malignancies or critical sepsis. As a result, the albumin component, while statistically significant on its own (AUC: 0.751), was insufficient to elevate the composite CALLY index beyond the performance of CRP alone. Taken together, these findings suggest that the CALLY index may be best suited for clinical conditions in which all three of its components undergo substantial and concurrent derangement, rather than for infections like brucellosis where CRP is the predominantly altered parameter [4,9,26,31].

Despite these findings, the CALLY index remains a conceptually valuable tool that reflects the interplay between inflammation, nutrition, and immunity. Its performance may differ in patient populations with a higher proportion of chronic or subacute brucellosis, where albumin depletion and CD4+ T-cell lymphocyte exhaustion are more pronounced [4]. Furthermore, the dynamic monitoring of the CALLY index during therapy warrants thorough elaboration. During prolonged triple-antibiotic regimens, the resolution of acute systemic inflammation (CRP drop), the recovery of physiological stress (albumin synthesis), and the restoration of cellular immunity (lymphocyte proliferation) occur at different rates. Therefore, serial evaluations of the CALLY index could theoretically capture this multiphasic recovery better than single markers. Future prospective studies focusing on the dynamic changes of the CALLY index could establish its role as an early indicator of therapeutic response, treatment failure, or impending relapse, particularly in subacute and chronic cases where immuno-nutritional deficits are more pronounced.

This study has several limitations. First, it was conducted at a single center with a retrospective design, which may introduce selection bias and limits the generalizability of the findings. External validation in independent cohorts from different endemic regions is warranted to confirm these results. Second, the relatively limited sample sizes in non-osteoarticular focal subgroups (e.g., neurological or cardiovascular involvements) prevented us from conducting subgroup-specific predictive analyses. Third, the predominance of acute-phase patients in our cohort (approximately 80%) may have attenuated the contributions of albumin and lymphocytes to the CALLY index. Consequently, subgroup analyses stratified by disease phase (acute vs. subacute/chronic) were not feasible due to the limited sample size of chronic cases. This should be explicitly recognized as a limitation that may affect the broader interpretation of our prognostic findings across different clinical stages of the disease. Fourth, the CALLY index formula has not been standardized across studies, and variations in the units used for its components may affect the comparability of CALLY values and optimal cut-off points across different populations. Fifth, a potential incorporation (workup) bias cannot be completely ruled out. Although the diagnosis of focal involvement was established strictly through objective imaging modalities (e.g., MRI, CT, ultrasonography) and microbiological criteria to ensure independence from laboratory parameters, the clinical decision to perform these imaging studies in real-world practice may have been partially influenced by elevated inflammatory markers (such as ESR or CRP). This could theoretically inflate the observed predictive performance of these biomarkers. Sixth, the comprehensive assessment of patient comorbidities and detailed nutritional status was limited. Finally, the laboratory parameters and the CALLY index were calculated only based on admission values; dynamic monitoring of these indices during the course of triple-antibiotic therapy could provide further insights into their utility in predicting treatment response or relapse [5,22,32].

In conclusion, this study is the first to evaluate the CALLY index in human brucellosis, demonstrating that it does not provide incremental diagnostic value over traditional markers for predicting focal complications at initial presentation. Our findings carry a clear, practice-relevant message: clinicians should not uncritically adopt novel composite biomarkers for early risk stratification when inexpensive, widely accessible, and statistically robust alternatives exist. We established that an elevated ESR remains the most powerful independent predictor of early focal complications, serving as a crucial triage tool to guide immediate radiological imaging. Nevertheless, while its acute-phase utility is limited, the CALLY index remains a valuable conceptual model reflecting the immuno-nutritional deficits of the disease. Its true clinical potential likely resides outside the acute diagnostic phase, holding promise for the dynamic monitoring of therapeutic responses and risk-stratification in chronic brucellosis. Prospective, multi-center studies focusing on serial measurements are warranted to determine the exact role of such composite biomarkers in comprehensive clinical decision-making.

## Figures and Tables

**Table 1:** Demographic and epidemiological characteristics of patients with brucellosis.

Characteristics	Focal Involvement (-) (n = 114)	Focal Involvement (+) (n = 89)	*p*-Value
Male, n (%)	75 (65.8)	57 (64.0)	0.796
Age, mean ± SD	46.38 ± 16.13	51.27 ± 15.37	0.029
Age, median (IQR)	47 (32.5–59.5)	53 (40.5–62)	
Exposure history, n (%)			
Animal husbandry	45 (39.5)	42 (47.2)	0.270
Contact with aborted animal materials	32 (28.1)	28 (31.5)	0.599
Consumption of unpasteurized food	106 (93.0)	86 (96.6)	0.255
History of fever, n (%)	37 (32.5)	49 (55.1)	0.001
Muscle and joint pain, n (%)	92 (80.7)	78 (87.6)	0.184
Headache, n (%)	6 (5.3)	12 (13.5)	0.041
Bacteremia, n (%)	6 (5.3)	8 (9.0)	0.299
Disease course, n (%)			
Acute phase	94 (82.5)	67 (75.3)	0.210
Subacute/chronic phase	20 (17.5)	22 (24.7)	

Note: Data are expressed as mean ± standard deviation (SD) or median (interquartile range, [IQR]) for continuous variables, and as frequencies and percentages (n, %) for categorical variables. Statistical comparisons between the non-focal (-) and focal (+) involvement groups were performed using Student’s *t*-test or Mann–Whitney U test for continuous variables, and Pearson’s Chi-square test or Fisher’s exact test for categorical variables, as appropriate. A *p*-value of <0.05 was considered statistically significant. Abbreviations: SD: Standard Deviation; IQR: Interquartile Range.

**Table 2:** Laboratory findings of patients with brucellosis.

Laboratory Findings	Focal Involvement (-) (n = 114)	Focal Involvement (+) (n = 89)	*p*-Value
ESR (mm/h), median (IQR)	16.5 (7–29)	43 (34–67)	<0.001
CRP (mg/L), median (IQR)	18 (9–30)	35 (20–68)	<0.001
Leukocyte (×10^9^/L), median (IQR)	6.4 (5.2–7.6)	7.2 (6.0–8.64)	0.003
Monocyte (×10^9^/L), median (IQR)	0.5 (0.4–0.7)	0.6 (0.4–0.8)	0.001
Lymphocyte (×10^9^/L), median (IQR)	2.15 (1.7–2.7)	2.0 (1.7–2.7)	0.615
Neutrophil (×10^9^/L), median (IQR)	3.5 (2.6–4.2)	4.1 (3.0–5.2)	0.006
Platelet count (×10^9^/L), mean ± SD	245.15 ± 67.13	300.24 ± 101.14	<0.001
Hemoglobin (g/dL), mean ± SD	13.74 ± 1.733	12.72 ± 1.489	<0.001
MPV, median (IQR)	8.55 (7.8–9.6)	8.2 (7.6–8.9)	0.007
RDW, median (IQR)	13.75 (13.0–14.8)	14.0 (13.2–15.5)	0.140
Albumin (g/L), median (IQR)	42 (39–44)	37 (35–40)	<0.001
ALT (U/L), median (IQR)	25 (17–41)	29 (18–54)	0.207
AST (U/L), median (IQR)	24 (20–39)	28 (20–51)	0.151
NLR, median (IQR)	1.56 (1.06–2.18)	1.82 (1.35–2.52)	0.019
PLR, median (IQR)	0.112 (0.092–0.140)	0.137 (0.099–0.177)	0.001
LMR, median (IQR)	4.25 (3.25–5.8)	3.48 (2.52–4.5)	0.001
CALLY index, median (IQR)	5.25 (3.03–10.92)	2.35 (1.00–4.43)	<0.001

Note: Data are expressed as mean ± standard deviation (SD) for normally distributed continuous variables (platelet count and hemoglobin), and as median (interquartile range, [IQR]) for non-normally distributed continuous variables. Statistical comparisons between the non-focal (-) and focal (+) involvement groups were performed using Student’s *t*-test for normally distributed data and the Mann–Whitney U test for non-normally distributed data. A *p*-value of <0.05 was considered statistically significant. Abbreviations: ALT: Alanine Aminotransferase; AST: Aspartate Aminotransferase; CALLY: C-reactive protein, Albumin, and Lymphocyte index; CRP: C-reactive Protein; ESR: Erythrocyte Sedimentation Rate; IQR: Interquartile Range; LMR: Lymphocyte-to-Monocyte Ratio; MPV: Mean Platelet Volume; NLR: Neutrophil-to-Lymphocyte Ratio; PLR: Platelet-to-Lymphocyte Ratio; RDW: Red Cell Distribution Width; SD: Standard Deviation.

**Table 3:** ROC Curve Analysis Summary.

Parameter	AUC	95% CI	*p*-Value	Cut-Off	Sensitivity	Specificity
ESR	0.839	0.783–0.896	<0.001	23.5	88.76%	69.30%
CRP	0.720	0.649–0.791	<0.001	26.5	64.40%	71.93%
CALLY index	0.728	0.658–0.798	<0.001	4.673	78.65%	57.89%
Hemoglobin	0.679	0.606–0.752	<0.001	13.95	83.15%	50.88%
Albumin	0.751	0.682–0.820	<0.001	39.5	71.91%	74.56%
MPV	0.610	0.532–0.687	0.007	9.15	80.90%	35.96%
NLR	0.592	0.513–0.671	0.025	1.54	69.66%	50.00%
PLR	0.635	0.558–0.713	0.001	0.128	55.06%	68.42%
LMR	0.641	0.565–0.718	0.147	3.586	57.30%	66.67%

Note: The Area Under the Curve (AUC) represents the overall discriminative ability of each biomarker. The optimal cut-off values were determined using the Youden index, which maximizes both sensitivity and specificity. Diagnostic metrics, including sensitivity and specificity, are presented as percentages corresponding to these calculated cut-off points. A *p*-value < 0.05 indicates a statistically significant discriminative value compared to a random classifier (AUC = 0.50). Abbreviations: AUC: Area Under the Curve; CALLY: C-reactive protein, Albumin, and Lymphocyte index; CI: Confidence Interval; CRP: C-reactive Protein; ESR: Erythrocyte Sedimentation Rate; LMR: Lymphocyte-to-Monocyte Ratio; MPV: Mean Platelet Volume; NLR: Neutrophil-to-Lymphocyte Ratio; PLR: Platelet-to-Lymphocyte Ratio; ROC, Receiver Operating Characteristic.

**Table 4:** Pairwise AUC Comparisons (DeLong Test).

Comparison	AUC Difference	95% CI of Difference	*p*-Value
ESR vs. Albumin	0.09087	0.0168–0.1649	0.016
ESR vs. CRP	0.12034	0.0476–0.1930	0.001
ESR vs. CALLY index	0.11443	0.0423–0.1866	0.002
Albumin vs. CRP	0.02947	−0.0598–0.1187	0.517
Albumin vs. CALLY index	0.02356	−0.0599–0.1071	0.580
CALLY index vs. CRP	0.00591	−0.0220–0.0338	0.678

Note: The table presents the pairwise statistical comparison of the discriminative performance of the most significant individual biomarkers and the CALLY index. The DeLong method was utilized to evaluate the statistical significance of the difference between the areas under two correlated Receiver Operating Characteristic (ROC) curves. An AUC difference with a 95% Confidence Interval (CI) that does not cross zero, accompanied by a *p*-value < 0.05, indicates a statistically significant difference in predictive capability between the two compared parameters. Abbreviations: AUC, Area Under the Curve; CALLY, C-reactive protein, Albumin, and Lymphocyte; CI, Confidence Interval; CRP, C-reactive Protein; ESR, Erythrocyte Sedimentation Rate; ROC, Receiver Operating Characteristic.

**Table 5:** Multivariate Logistic Regression Models for Predicting Focal Involvement.

Variables	Model 1 (Enter) OR (95% CI)	*p*-Value	Model 2 (Enter) OR (95% CI)	*p*-Value
CRP	0.998 (0.984–1.011)	0.732	-	-
Albumin	0.934 (0.864–1.011)	0.090	-	-
LogCALLY	-	-	0.753 (0.526–1.079)	0.122
ESR	1.046 (1.024–1.067)	<0.001	1.042 (1.022–1.062)	<0.001
Hemoglobin	0.837 (0.646–1.085)	0.179	0.797 (0.626–1.014)	0.065
MPV	0.851 (0.635–1.139)	0.278	0.826 (0.619–1.102)	0.194
Age	0.998 (0.976–1.021)	0.866	1.000 (0.978–1.023)	0.994
Gender (Male)	0.543 (0.235–1.254)	0.153	0.593 (0.262–1.344)	0.210

Note: The table presents two distinct multivariate models constructed using the standard ‘Enter’ method (simultaneous inclusion of all selected variables). To prevent inherent mathematical coupling and multicollinearity, the components of the CALLY index (CRP and Albumin) were exclusively evaluated in Model 1, whereas the logarithmic transformation of the CALLY index (LogCALLY) was exclusively evaluated in Model 2. The Odds Ratio (OR) indicates the multiplicative change in the odds of focal involvement for each one-unit increase in continuous variables, or for the specified category in categorical predictors (e.g., Male). Model performance and calibration metrics: Model 1: Nagelkerke $R^{2}$ = 0.396, overall classification accuracy = 78.8%, Hosmer–Lemeshow goodness-of-fit test p < 0.001. Internal validation was confirmed via bootstrapping (1000 resamples). Model 2: Nagelkerke $R^{2}$ = 0.404, overall classification accuracy = 77.8%, Hosmer–Lemeshow goodness-of-fit test p = 0.001. Internal validation was confirmed via bootstrapping (1000 resamples). Abbreviations: CALLY, C-reactive protein, Albumin, and Lymphocyte; CI, Confidence Interval; CRP, C-reactive Protein; ESR, Erythrocyte Sedimentation Rate; LogCALLY, Logarithmic transformation of the CALLY index; MPV, Mean Platelet Volume; OR, Odds Ratio.

## Data Availability

The data presented in this study are available on request from the corresponding author.

## Abbreviations

The following abbreviations are used in this manuscript:

ALT	Alanine Aminotransferase
AST	Aspartate Aminotransferase
AUC	Area Under the Curve
CALLY	C-reactive protein, Albumin, and Lymphocyte
CI	Confidence Interval
CRP	C-reactive Protein
CSF	Cerebrospinal Fluid
CT	Computed Tomography
ESR	Erythrocyte Sedimentation Rate
Hgb	Hemoglobin
IQR	Interquartile Range
LMR	Lymphocyte-to-Monocyte Ratio
LogCALLY	Base-10 logarithm of the CALLY index
MPV	Mean Platelet Volume
MRI	Magnetic Resonance Imaging
NLR	Neutrophil-to-Lymphocyte Ratio
OR	Odds Ratio
PLR	Platelet-to-Lymphocyte Ratio
RDW	Red Cell Distribution Width
ROC	Receiver Operating Characteristic
SD	Standard Deviation
STA	Standard Tube Agglutination
USG	Ultrasonography

## References

[1] World Health Organization (WHO) Brucellosis. https://www.who.int/news-room/fact-sheets/detail/brucellosis.

[2] Franc K.A., Krecek R.C., Häsler B.N., Arenas-Gamboa A.M. (2018). Brucellosis remains a neglected disease in the developing world: A call for multidisciplinary action. BMC Public Health.

[3] Laine C.G., Johnson V.E., Scott H.M., Arenas-Gamboa A.M. (2023). Global estimate of human brucellosis incidence. Emerg. Infect. Dis..

[4] Zhang X., Zheng C., Chen X., Zhang L., Pang L., Zhang Y. (2025). The influential factors on clinical outcomes of focal brucellosis: A retrospective cohort study. Acta Trop..

[5] Demirdal T., Sen P. (2020). Risk factors for focal involvement in brucellosis. Diagn. Microbiol. Infect. Dis..

[6] Copur B., Sayili U. (2022). Laboratory and clinical predictors of focal involvement and bacteremia in brucellosis. Eur. J. Clin. Microbiol. Infect. Dis..

[7] Xu N., Dong X., Yao Y., Guan Y., Chen F., Zheng F., Fan W. (2020). Improved early detection of focal brucellosis complications with anti-Brucella IgG. J. Clin. Microbiol..

[8] Özer-Balin Ş., Bozkurt T., Öztürk-Kaygusuz T., Akbulut A., Demirdağ K. (2024). Evaluation of brucellosis cases and complications. Klimik Derg..

[9] Mancinetti F., Guazzarini A.G., Gaspari M., Croce M.F., Serra R., Mecocci P., Parnetti L. (2025). Integrating Nutrition, Inflammation, and Immunity: The CALLY Index as a Novel Prognostic Biomarker in Acute Geriatric Care. Nutrients.

[10] Liu X.Y., Zhang X., Zhang Q., Ruan G.T., Liu T., Xie H.L., Wang Z.X. (2023). The value of CRP-albumin-lymphocyte index (CALLY index) as a prognostic biomarker in patients with non-small cell lung cancer. Support. Care Cancer.

[11] Xie C., Sun P., Zhang M., Fan H., Li Z., Tong X. (2026). The prognostic value of the CALLY index in sepsis: A systematic review and meta-analysis. Front. Med..

[12] Sarıdaş A., Çetinkaya R. (2025). The Prognostic Value of the CALLY Index in Sepsis: A Composite Biomarker Reflecting Inflammation, Nutrition, and Immunity. Diagnostics.

[13] Orta H., Aydin C., Demirkiran A., Orta Z. (2026). Prognostic value of the CALLY index in predicting mortality in patients with infective endocarditis. Biomark. Med..

[14] Pan Y., Liu Z., Tu R., Feng X., Yu F., Wei M., Zhang L. (2025). The value of the CRP-albumin-lymphocyte index (CALLY index) as a prognostic biomarker in acute ischemic stroke. Sci. Rep..

[15] Dadar M., Sadooghi N., Ansari F., Hashemi S.A., Moghaddam H.G., Panahi Y., Jafari S. (2025). Risk factors for brucellosis relapse and focal complications: A nine-year multicenter study in an endemic region. Int. J. Infect. Dis..

[16] Feng Q., Ha X., Lin T., Guo W., Song Y. (2023). Brucella cultures characteristics, clinical characteristics, and infection biomarkers of human Brucellosis. J. Infect. Public Health.

[17] Spernovasilis N., Karantanas A., Markaki I., Konsoula A., Ntontis Z., Koutserimpas C., Alpantaki K. (2024). Brucella Spondylitis: Current Knowledge and Recent Advances. J. Clin. Med..

[18] Shi Q.N., Qin H.J., Lu Q.S., Li S., Tao Z.F., Fan M.G., Wang Y.P. (2024). Incidence and warning signs for complications of human brucellosis: A multi-center observational study from China. Infect. Dis. Poverty.

[19] Kuruoglu T., Sensoy L., Atilla A., Temocin F., Gur D., Tanyel E. (2023). Evaluation of risk factors for the development of bacteremia and complications in patients with brucellosis: Is it possible to predict the clinical course?. J. Infect. Dev. Ctries..

[20] Markanday A. (2015). Acute Phase Reactants in Infections: Evidence-Based Review and a Guide for Clinicians. Open Forum Infect. Dis..

[21] Kayaaslan B., Bastug A., Aydin E., Akinci E., But A., Aslaner H., Bodur H. (2016). A long-term survey of brucellosis: Is there any marker to predict the complicated cases?. Infect. Dis..

[22] Jin M., Fan Z., Gao R., Li X., Gao Z., Wang Z. (2023). Research progress on complications of Brucellosis. Front. Cell. Infect. Microbiol..

[23] Zheng R., Xie S., Niyazi S., Lu X., Sun L., Zhou Y., Cao L. (2018). Meta-Analysis of the Changes of Peripheral Blood T Cell Subsets in Patients with Brucellosis. J. Immunol. Res..

[24] Franco M.P., Mulder M., Gilman R.H., Smits H.L. (2007). Human brucellosis. Lancet Infect. Dis..

[25] de Figueiredo P., Ficht T.A., Rice-Ficht A., Rossetti C.A., Adams L.G. (2015). Pathogenesis and immunobiology of brucellosis: Review of Brucella-host interactions. Am. J. Pathol..

[26] Iida H., Tani M., Komeda K., Nitta H., Mouri T., Sato T., Okinaga H. (2022). Superiority of CRP-albumin-lymphocyte index (CALLY index) as a non-invasive prognostic biomarker after hepatectomy for hepatocellular carcinoma. HPB.

[27] Pang H., Dai F., Zhao Z., Chen X., Yan M., Chen L., Zhang L. (2026). Clinical significance of the pretreatment C-reactive protein-albumin-lymphocyte (CALLY) index in colorectal cancer patients: A meta-analysis. BMC Cancer.

[28] Yılmaz E., Ak R. (2025). Evaluation of the C-reactive protein-albumin-lymphocyte (CALLY) index as a prognostic marker in patients with sepsis. BMC Emerg. Med..

[29] Mantovani A., Garlanda C. (2023). Humoral innate immunity and acute-phase proteins. N. Engl. J. Med..

[30] Zheng R., Xie S., Zhang Q., Cao L., Niyazi S., Lu X., Sun L. (2019). Circulating Th1, Th2, Th17, Treg, and PD-1 levels in patients with brucellosis. J. Immunol. Res..

[31] Jellinge M.E., Henriksen D.P., Hallas P., Brabrand M. (2014). Hypoalbuminemia is a strong predictor of 30-day all-cause mortality in acutely admitted medical patients: A prospective, observational, cohort study. PLoS ONE.

[32] Buzgan T., Karahocagil M.K., Irmak H., Baran A.I., Karsen H., Evirgen O., Akdeniz H. (2010). Clinical manifestations and complications in 1028 cases of brucellosis: A retrospective evaluation and review of the literature. Int. J. Infect. Dis..

